# Synthesis, Spectral Characterization, and Antiproliferative Studies of Mixed Ligand Titanium Complexes of Adamantylamine

**DOI:** 10.1155/2014/142828

**Published:** 2014-02-27

**Authors:** Raj Kaushal, Nitesh Kumar, Ashun Chaudhary, Saroj Arora, Pamita Awasthi

**Affiliations:** ^1^Department of Chemistry, National Institute of Technology, Hamirpur, Himachal Pradesh 177005, India; ^2^Department of Botanical and Environmental Sciences, Guru Nanak Dev University, Amritsar, Punjab 143005, India

## Abstract

Titanium complexes have been synthesized by the reaction between titanium tetrachloride (TiCl_4_), respective bidentate ligand [4,4′ -dimethoxy-2,2′ -bipyridine (bpome), 6,6′-dimethyl-2,2′-bipyridine (dpme), 1,2-diaminocyclohexane (dach), 1,10-phenanthroline (phen), and benzoylacetone (bzac)], and adamantylamine (ada) in 1 : 2 : 2 molar ratios, respectively. The structure of synthesized complexes was confirmed using elemental analysis, FTIR, UV-visible, ^1^H NMR, and mass spectrometry techniques. The nanocrystalline nature of complexes was confirmed by powder XRD study. The complexes were evaluated for cytotoxic potential in HeLa (cervical), C6 (glioma), and CHO (Chinese hamster ovarian) cell lines. The complex E was found to be more effective cytotoxic agent against HeLa cell line with an IC_50_ value of 4.06 µM. Furthermore, the effect of synthesized complexes was studied on different stages of the cell cycle in CHO cells. All complexes exhibited the dose dependent increase in cytotoxicity. The results have shown an increase in sub-G_0_ population with increase in concentration which is an indicative measure of apoptosis.

## 1. Introduction

The discovery of cisplatin, a metal (platinum) based anticancer drug by Rosenberg et al. in 1965, has created interest in the development of metal based anticancer drugs [1–3]. The effect of transition metal complexes, other than platinum such as ruthenium [4–8], palladium [9–13], gold [14, 15], and titanium [16–25] has also been studied on several cancer cell lines. In addition to cisplatin, many other platinum based drugs,namely, carboplatin, oxaliplatin, tetraplatin, and satraplatin [3], and nonplatinum based drugs, namely, budotitane, titanocene dichloride [16], NAMI-A, KP1019 [26–29], and auranofin [14] have shown remarkable results. Out of these, the first nonplatinum anticancer drugs were budotitane and titanocene dichloride which are titanium based drugs [16]. These titanium complexes offer an alternative to chemotherapy, although these complexes do not follow a mechanism similar to that of other metal complexes. Previous studies have revealed that titanium compounds are effective against those cell lines which are resistant to platinum based drugs and kill the cancer cells through apoptosis. It has also been confirmed that lability of ligand is not a mandatory condition for a compound to show cytotoxicity [30], but other ligand properties have been found to be necessary for this activity [31]. It is well established that ligands having electron donating atom(s) show increased cytotoxicity due to enhanced coordination capacity [32, 33]. Since few efforts have been made towards the synthesis and use of titanium complexes as chemotherapeutic agents, this is an important area of research. In the present work, we report the synthesis, structural characterization, and antiproliferative potential of some of titanium complexes.

## 2. Experimental Section

### 2.1. Materials and Methods

Ligands and titanium tetrachloride used were obtained from Sigma Aldrich. All the solvents were of AR grade (Merck) and purified by standard procedure before use and stored over 4 Å molecular sieves. Purity of ligands was checked by checking their melting points. Elemental analyses were performed by using Perkin-Elmer, Series 2400. The UV-visible spectra of the complexes were recorded on Perkin Elmer Lambda 750 in the range of 200–800 nm and FTIR Spectra were recorded from 4000–200 cm^−1^on Perkin Elmer 1600. The mass spectrum was recorded by using the electron spray ionization technique on Waters Micromas Q Tof Micro. ^1^H NMR Spectra were recorded on Brucker Avance 400 MHz spectrometer. Crystalline nature of the complexes has been confirmed by powder XRD technique on Philips 1710 X-ray diffractometer.

### 2.2. Synthetic Procedures (A–E)

#### 2.2.1. Synthesis of Bis(adamantylamino)bis-(4,4′-dimethoxy-2,2′-bipyridyl)titanium(II), Ti(ada)_2_(bpome)_2_, **(A)**

To a colorless solution of 4,4′-dimethoxy-2,2′-bipyridyl (0.45 g, 2.1 mmol) in 25 mL of toluene, a pale yellow colored solution of titanium tetrachloride (0.2 g, 1.05 mmol) in 25 mL of toluene was added dropwise with continuous stirring under ice cold conditions. The reaction mixture was stirred for 2 h followed by refluxing for 10 h till the evolution of chlorine gas ceased. The evolution of chlorine gas was checked by passing the gas through a potassium iodide solution which results in reddish brown color of potassium iodide due to liberation of iodine. After removing solvent through vacuum distillationcompound was dried under vacuum. A light yellow colored solid compound [TiCl_2_(bpome)_2_] was obtained. Yield: 0.5 g (86.2%). Now, to a solution of TiCl_2_(bpome)_2_ (0.5 g, 0.91 mmol) in 25 mL of toluene, adamantylamine (0.27 g, 1.81 mmol) in 25 mL of toluene was added dropwise with continuous stirring. The reaction mixture was stirred for 2 h and refluxed for 14 h till the evolution of HCl gas ceased. The evolution of HCl gas was confirmed by passing the gas through an ammonia solution which results in white dense fumes of ammonium chloride. The excess solvent was removed by vacuum distillation and the compound was washed with petroleum ether. The compound was dried under vacuum. A cream colored solid powder was obtained which was recrystallised in methanol. Yield: 0.45 g (64.28%). TiC_44_H_54_N_6_O_4_: elemental anal. Calcd (%): C 67.67, H 7.17, N 10.76; found (%): C 66.71, H 7.05, N 10.52.FTIR (KBr, cm^−1^) $\overset{-}{\nu }$: 3388 (NH Stretching), 3015 (aromatic CH stretching), 2927 (CH stretching), 1629 (C=C stretching), 1522 (C=N stretching), 1449 (NH bending) 1313, 1229 (CH bending), 906, 809 (CH out of plane deformation), 452 (Ti–N stretching). ^1^H NMR (DMSO, 400 MHz): adamantylamine **δ**, ppm = 2.1 (s, NH), 1.86 (d, ^*3*^ *J* = 1.96 Hz, CH_2_ protons), 1.66, 1.59, (dd, ^*3*^ *J* = 12.36, 29.6 Hz CH protons). 4,4′-Dimethoxy-2,2′-bipyridine **δ**, ppm = 8.66 (d, ^*3*^ *J* = 6.16 Hz, 4H, H^6^), 7.96 (s, 4H, H^3^), 7.37 (d, ^*3*^ *J* = 8 Hz, 4H, H^5^), 4.09 (s, 12H, OCH_3_).

#### 2.2.2. Synthesis of Bis(adamantylamino)bis-(6,6′-dimethyl-2,2′-bipyridyl)titanium(II), Ti(ada)_2_(dpme)_2_, **(B)**

The complex was synthesized in accordance to the procedure used for complex A. Yield: 0.4 g (84.38%). TiC_44_H_54_N_6_: elemental anal. Calcd (%): C 73.70, H 7.81, N 11.72; found (%): C 73.91, H 7.94, N 11.54. FTIR (KBr, cm^−1^) $\overset{-}{\nu }$: 3336 (NH stretching), 3071 (aromatic CH stretching), 2925 (CH stretching), 1644 (C=C stretching), 1506 (C=N stretching), 1441 (NH bending) 1271, 1117 (CH bending), 906, 801 (CH out of plane deformation), 404 (Ti–N stretching). ^1^H NMR (DMSO, 400 MHz): adamantylamine **δ**, ppm = 2.12 (s, NH), 1.91 (d, ^*3*^ *J* = 2.12 Hz, CH_2_ protons), 1.69, 1.64, (dd, ^*3*^ *J *= 12.6, 22.6 Hz CH protons). 6,6′-Dimethyl-2,2′-bipyridine **δ**, ppm = 8.5 (d, ^*3*^ *J* = 7.96 Hz, 4H, H^5^), 8.23 (t, ^*3*^ *J* = 7.8, 7.8 Hz 4H, H^4^),7.68 (d, ^*3*^ *J* = 7.8 Hz, 4H, H^3^), 2.89 (s, 12H, CH_3_).

#### 2.2.3. Synthesis of Bis(adamantylamino)bis-(1,2-diaminocyclohexane)titanium(II), Ti(ada)_2_(dach)_2_, **(C)**

The complex C was synthesized similarly to complex A. Yield = 0.48 g (87.2%). TiC_32_H_58_N_6_: elemental anal. Calcd (%): C 66.63, H 10.41, N 14.57; found (%): C 65.77, H 10.23, N 14.72. FTIR (KBr, cm^−1^) $\overset{-}{\nu }$: 3379 (NH stretching), 2925, 2900 (CH stretching), 1595 (C–N stretching), 1522 (NH bending), 1360, 1311 (CH bending), 1084, 1020 (CH out of plane deformation), 444 (Ti–N stretching). ^1^H NMR (D_2_O, 400 MHz): adamantylamine **δ**, ppm = 2.09 (s, NH), 1.7, ^*3*^ *J* = 4 (d, CH_2_ protons), 1.63, 1.55, (dd, ^*3*^ *J* = 12.52, 36.76 CH protons). 1,2-Diaminocyclohexane **δ**, ppm =3.63 (t, ^*3*^ *J* = 4.8, 4.64 CH), 3.3(m, NH_2_), 2.01, 1.76, 1.68, 1.3 (H^3^, H^6^, H^4^, H^5^).

#### 2.2.4. Synthesis of Bis(adamantylamino)bis-(1,10-phenanthroline)titanium(II), Ti(ada)_2_(phen)_2_, **(D)**

The procedure described above for complex A was followed for the synthesis of complex D. Yield: 0.42 g (87.5%). TiC_44_H_46_N_6:_ elemental anal. Calcd (%): C 74.55, H 6.77, N 11.85; found (%): C 74.46, H 6.42, N 11.52. FTIR (KBr, cm^−1^) $\overset{-}{\nu }$: 3412 (NH stretching), 3039 (aromatic CH stretching), 2927 (CH stretching), 1612 (C=C stretching), 1514 (N–H bending), 1449 (C=N stretching), 1368, 1319 (CH bending), 1084 (CH out of plane deformation), 411 (Ti–N stretching).^1^H NMR (D_2_O, 400 MHz): adamantylamine **δ**, ppm = 2.04 (s, NH), 1.75 (d, ^*3*^ *J* = 2.52, CH_2_ protons), 1.61, 1.55, (dd, ^*3*^ *J* = 24.8 CH protons). 1,10-Phenanthroline **δ**, ppm = 8.56 (d, ^*3*^ *J* = 7.43, 4H, H^2^& H^9^), 7.7 (s, 4H, H^5^& H^6^), 7.4 (d, ^*3*^ *J* = 9.28, 4H, H^4^& H^7^), 6.8 (dd, ^*3*^ *J* = 28, 40, 4H, H^3^& H^8^).

#### 2.2.5. Synthesis of Bis(adamantylamino)bis(benzoylacetonato)titanium(IV), Ti(ada)_2_(bzac)_2_, **(E)**

The procedure for the synthesis of complex A was followed for the preparation of complex E. However, there was evolution of HCl gas in both the steps. Yield: 0.43 g (86%). TiC_40_H_48_N_2_O_2_: elemental anal. Calcd (%): C 71.61, H 7.45, N 4.17; found (%): C 71.70, H 7.27, N 4.10. FTIR (KBr, cm^−1^) $\overset{-}{\nu }$: 3379 (NH stretching), 2933, 2866 (CH stretching), 1612 (C=O stretching), 1522 (C=C stretching), 1449 (NH bending), 1109, 1004 (C–H bending), 557 (Ti–O stretching), 427 (Ti–N stretching). ^1^H NMR (D_2_O, 400 MHz): adamantylamine **δ**, ppm = 2 (s, NH), 1.71 (d, ^*3*^ *J* = 2.56, CH_2_ protons), 1.58, 1.49, (dd, ^*3*^ *J* = 12.56, 12.08 CH protons). Benzoylacetone **δ**, ppm C_6_H_5_: *δ* = 8.08 (d, ^*3*^ *J* = 7.52, 4H, H^2^and H^6^), 7.96 (t, ^*3*^ *J* = 7.16, 4.28, 4H, H^3^and H^5^), 7.6 (t, ^*3*^ *J* = 7.64, 7.28, 2H, H^4^), 3.92 (s, CH protons), 2.5 (s, CH_3_ protons).

### 2.3. Cytotoxicity Studies

#### 2.3.1. Cell Lines and Culture

The cytotoxic studies of synthesized complexes were performed on HeLa (cervical cancer cell line), C6 (glioma), and CHO (Chinese hamster ovarian) cell line. The cells were grown in Dulbecco's Modified Eagle's Medium (DMEM) containing fetal calf serum (FCS) (10%), penicillin (100 units/mL), and streptomycin (100 *μ*g/mL) at 37°C with 90% humidity and 5% CO_2_. The complexes were dissolved in dimethyl sulphoxide (DMSO) to prepare the solutions of different concentrations. The selected cell lines were treated with these solutions to calculate the IC_50_ values by using MTT (3-(4,5-dimethylthiazol-2-yl)-2,5-diphenyltetrazolium bromide) assay while control cells received only DMSO.

#### 2.3.2. MTT Assay

The growth inhibitory effect of newly synthesized titanium complexes on HeLa, C6, and CHO cells was determined by MTT assay [34]. For this, cells were supplemented in complete growth medium to get 1 × 10^5^ cells/mL and 100 *μ*L of cell suspension per well was seeded in tissue culture plate. The assay was carried out in 96 well plates in triplicate in which cells were treated with three different concentrations of complexes and incubated for 12 h in CO_2_ incubator. Thereafter, 20 *μ*L of freshly prepared MTT solution, 5 mgmL^−1^ in PBS (phosphate buffered saline) after sterile filtering, was added to each well. Now, culture plates were stirred at 150 rpm for 5 min to thoroughly mix MTT into the media. The plates were further incubated for 4 h at 37°C to allow metabolization of MTT. MTT formazan crystals were resuspended in 100 *μ*L of DMSO and plates were stirred for 20 min in order to dissolve formazan crystals and optical density was measured at 570 nm. The phase contrast imaging was done by using Nikon Eclipse TS100 inverted microscope.

#### 2.3.3. Cell Cycle Analysis

1 × 10^6^ cells/dish well was plated in 24 well plates which were allowed for adheration for 6 h and then treated with complexes at three different concentrations. After 24 h of treatment, cells were harvested from the plate. The cell suspension having 1 × 10^6^ cells was centrifuged and the resultant cell pellet was resuspended in phosphate buffered saline (1 mL) solution. The cells were fixed in ice cold 70% ethanol and stained with propidium iodide followed by analysis on the FL-2 channel by BD Accuri C6 flow cytometer (BD Biosciences Immunocytometry Systems, San Jose, CA). DNA content histograms and cell cycle phase distributions were modeled from at least 15,000 single events.

## 3. Results and Discussion

**Scheme 1:**
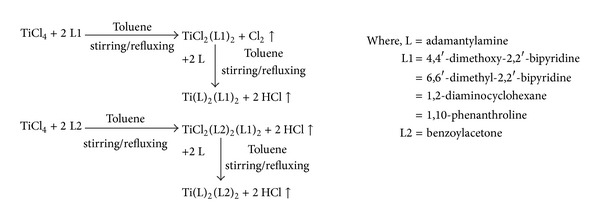
General synthetic route for mixed ligand titanium complexes of adamantylamine.

Synthesis of titanium complexes was carried out in two steps. In the first step, titanium tetrachloride reacted with respective bidentate ligand, that is, 4,4′-dimethoxy-2,2′-bipyridine, 6,6′-dimethyl-2,2′-bipyridine, 1,2-diaminocyclohexane, 1,10-phenanthroline, and benzoylacetone which are coligands in 1 : 2 molar ratio under continuous stirring and refluxing by using toluene as a solvent. There was evolution of chlorine gas during the course of reaction. In the next step, respective titanium complexes reacted with main ligand (adamantylamine) in 1 : 2 molar ratio in the same solvent, which results in evolution of HCl gas [35] as shown in Scheme 1. Elemental analysis, that is, Titanium and chlorine estimation, was performed to check the composition by gravimetric and Volhard's method, respectively, and molecular weight was determined by Rast's camphor method (Table 1). The analytical data and spectroscopic characterization of complexes confirm the proposed structure of complexes. The proposed structure of complexes and their corresponding ligands has been shown in Table 2.

### 3.1. FTIR Spectra

The bands of FTIR were assigned by comparing the spectra of complexes with those of free ligands and were shown in Table 3. From the spectra of complexes, we have found that wave number of ${\overset{-}{\nu }}_{\text{C–H}}$ band appearing around 2900–3000 cm^−1^ does not change much although the intensity of the band changes and gets weaker upon complexation with titanium metal. The absorption band due to ${\overset{-}{\nu }}_{\text{C=C}}$ stretching at 1595, 1578, and 1603 cm^−1^ in the bidentate ligand of complexes A, B, and D gets shifted to 1629, 1644, and 1612 cm^−1^ (Table 3). The shift may be due to reduction in electron density after an increase in conjugation caused by complexation with titanium metal [36]. In previous studies, it has been observed that three factors, namely, field effect, steric effect, and ring strain, can cause shift in vibrational frequencies of complexes. Due to field effect [37], the value of force constant gets changed and there is change in vibration frequencies, due to steric effect [36], the conjugation in the complex is not completed which results in a shift in absorption frequencies to higher wave number, and due to ring strain in the molecule, more energy is required for vibration of bonds which results in shift of band towards higher wave number. The ring breathing vibration (around 800–900 cm^−1^) having more intensity gets shifted to higher wave number in complexes (around 1000 cm^−1^). All these changes can be assigned to the coordinated nature of bidentate ligand through nitrogen atoms [12, 13]. The band formed around 3350–3400 cm^−1^ due to N–H stretching of adamantylamine ring, while the occurrence of a strong band in the region 1600–1580 cm^−1^ in complex E may be assigned to stretching modes of ${\overset{-}{\nu }}_{\text{C=O}}$ in benzoylacetone ligand. In complex E carbonyl groups are involved in bonding with the metal ion which is further supported by the appearance of an intense band at ~557 cm^−1^ assignable to ${\overset{-}{\nu }}_{\text{M–O}}$ vibration. Appearance of new bands at 452, 404, 444, 411, and 427 cm^−1^ in complexes A, B, C, D, and E shows that ligands are coordinated to the metal atom through nitrogen [35, 38] and the absence of bands in the region 385–340 cm^−1^ due to ${\overset{-}{\nu }}_{\text{Ti–Cl}}$ bond in all complexes indicates the complete removal of chloride ions [39].

### 3.2. UV-Visible Spectra

**Figure 1:**
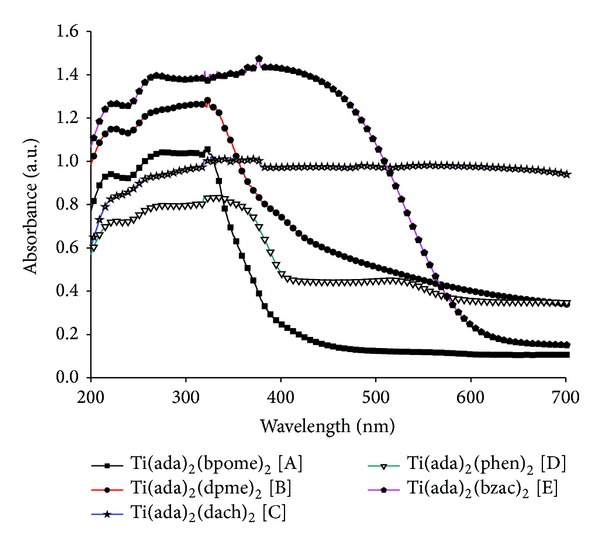
Electronic spectra of titanium complexes.

The UV-visible spectra of the complexes (Figure 1) and ligands were recorded from a solid sample by using diffuse reflectance technique. The transitions observed in the UV-visible spectrum of complexes were due to intraligand charge transfer. The transition around 320–325 nm can be attributed to *n* → π* transition in complexes A, B, D, and E get shifted to lower wavelength after coordination. However bands due to *π* → π* around 240–245 nm remain almost at the same position even after coordination. Since in complex C both the ligands are of cyclic nature, so there is no possibility of these transitions.

### 3.3. ^1^H NMR Study

The ^1^H NMR spectra of the complexes are consistent with the structures proposed in the reaction scheme. We find that bidentate ligands of synthesized complexes show a considerable downfield shift of protons after complexation with titanium. This shift may be due to transfer of electron density from ligand protons to the metal atom [35, 40]. However, protons of adamantylamine in all complexes appearing around 1.2–2.12 ppm show a marginal chemical shift. The cyclic aliphatic nature of both the ligands in complex C creates complications in the spectrum as the peaks corresponding to these falls almost in the same region. However, the integration of signals in all spectra supports the formation of proposed complexes.

### 3.4. Mass Spectra

The structure of complexes was further confirmed by recording electron spray mass spectrum. The complex A showed base peak at *m/z* = 152 due to C_10_H_17_N fragment ion and 4,4′-dimethoxy-2,2′-bipyridine ligand in the complex showed peak at *m/z* = 217 with relative intensity of 25%. We find that this complex also shows a peak at *m/z* = 478 due to TiC_24_H_24_N_4_O_4 _fragment ion. In case of complex B, one peak at *m/z* = 185 due to C_12_H_12_N_2_ fragment ion and another peak at *m/z* = 152 due to C_10_H_17_N fragment ion were found with a relative intensity of 18%. In complex C, peaks were formed at *m/z* = 98, 115, 230 due to C_6_H_12_N, C_6_H_14_N_2_, and TiC_10_H_19_N_2_ fragment ions. The complex D shows peaks at *m/z* = 304 and 335 due to TiC_16_H_22_N_3 _and TiC_16_H_22_N_3_ fragment ions_._ In addition to these peaks, complex D shows a molecular ion peak at *m/z* = 708 with very low intensity. In complexes C, D, and E, formation of the base peak takes place due to C_10_H_17_N fragment ion at *m/z* = 152. The complex E, in addition to base peak, also shows fragment ion peaks, in which one peak is formed at *m/z* = 401 due to TiC_22_H_32_N_2_O_2_ fragment ion. The complexes A, B, C, and E show their molecular ion peaks at *m/z* = 780, 716, 577, and 670 indicating the formation of complexes. The existence of these different fragment ion peaks, base peaks, and molecular ion peaks supports the stoichiometric formulation of synthesized complexes [35].

### 3.5. Powder XRD Study

**Figure 2:**
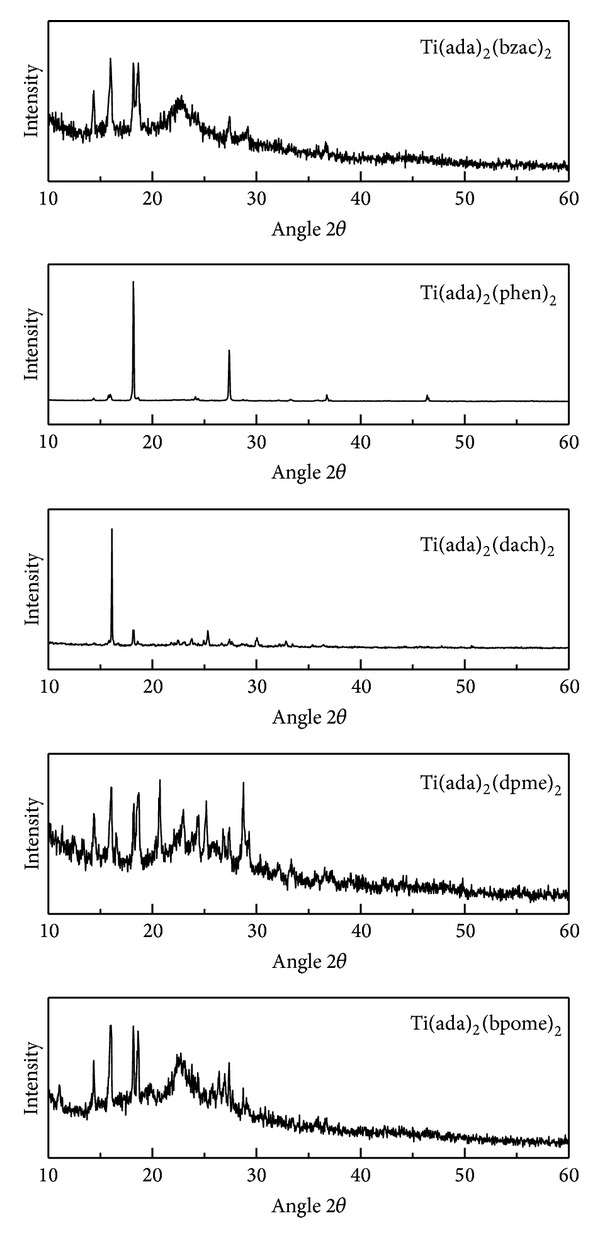
Powder XRD pattern of titanium complexes.

The powder X-ray diffraction study was performed to understand the lattice structure of the complexes.  Figure 2 shows XRD pattern obtained for all the complexes with well-defined peaks in these patterns which indicate the crystalline nature of complexes. Scherrer's equation D = (*λ* × 0.9)/(*β* × *Cos*⁡*θ*) [35, 41], with D as the crystallite size of (h k l) planes, *λ* as the wavelength of incident radiation (CuK*α*, 1.54 Å), and *β* as full width half maximum (FWHM), was used to calculate the crystallite size of complexes. The calculated crystallite size for complexes A, B, C, D, and E was 69, 26.5, 19.1, 115, and 76.6 nm, respectively, which falls in nanorange. Unit cell parameter of the complexes has been calculated by using Powder X software [42] and the results are summarized in Table 4. We have also observed that as the crystallite size decreases, peaks become broader as seen in Figure 2. On the basis of these different spectroscopic techniques, that is, UV-visible, FTIR, ^1^H NMR, and mass spectrometry, an octahedral geometry may be proposed for the synthesized titanium complexes [43].

### 3.6. MTT Assay

**Figure 3:**
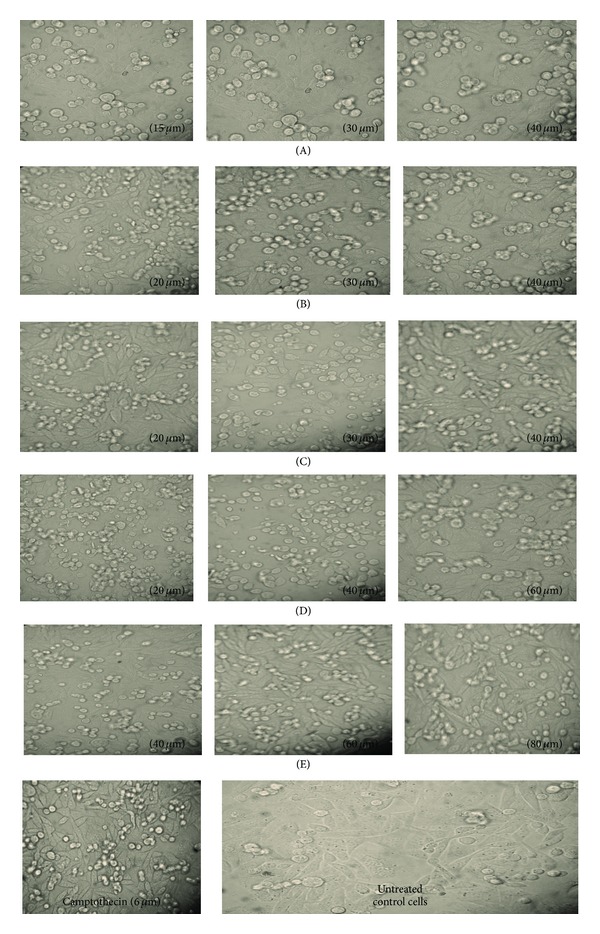
Morphology of CHO cells at different concentrations of titanium complexes.

The IC_50_ values were calculated by using best fit regression model and results have been tabulated in Table 5. The change in morphological features was observed at different concentrations of complexes, which indicates that such change in morphology is dose dependent as shown in Figure 3. The phase contrast imaging was done with a Nikon microscope at 40x after harvesting stage which clearly shows the formation of small apoptotic bodies, rounding of cells, shrinkage of cells, and plasma membrane blebbing. From the calculated IC_50_ values, it has been observed that complex E with benzoylacetone ligand shows (4.06 *μ*M) better activity than other complexes against the HeLa cell line, which is even better than known anticancer drug camptothecin as seen in Table 5. But ligands were found not much effective against the tested cancer cell lines. The IC_50_ values of main ligand, that is, adamantylamine along with its complexes, has been shown in Table 5. Complexes A, C, and D with 4,4′-dimethoxy-2,2′-bipyridine, 1,2-diaminocyclohexane, and 1,10-phenanthroline ligand shows good activity against all the tested cell lines which may be due to the presence of electron withdrawing nature of methoxy group, cyclic nature of 1,2-diaminocyclohexane, and aromatic nature of 1,10-phenanthroline ligand. However, complex B with 6,6′-dimethyl-2,2′-bipyridine was not found much effective against HeLa and C6 cell lines, which may be due to the presence of electron donating methyl groups in the ligand. So, it could be summarized that electron withdrawing group present in ligand as well as cyclic and aromatic nature of ligand are responsible for the cytotoxicity of titanium complexes.

### 3.7. Cell Cycle Analysis Using Propidium Iodide

**Figure 4:**
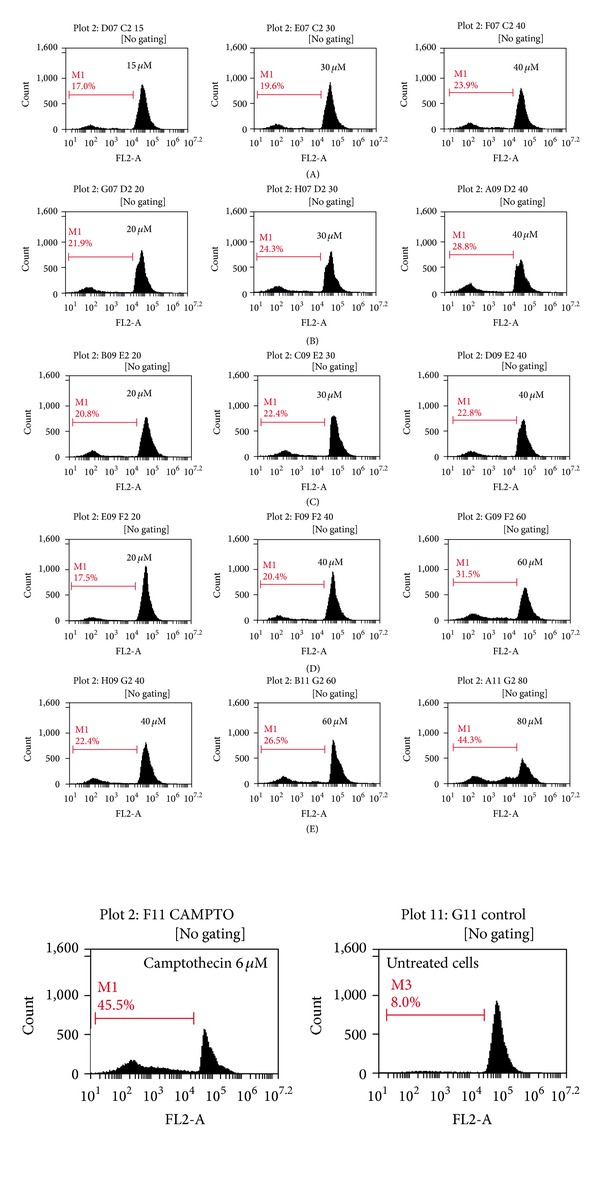
Cell cycle analysis of CHO cells exposed to different concentrations of titanium complexes that is, below IC50, near IC50, and above IC50 on flow cytometer by staining with propidium iodide.

For cell cycle analysis, CHO cells were treated with the complexes at three concentrations almost near to their IC_50_ values which caused the decrease in the number of cells with an increase in dose due to induction of apoptosis. It has been observed that all complexes increases cells in hypo-diploid cells of cell cycle and also increased the cell death with increase in concentration. Among all the complexes, complex E having benzoylacetone ligand showed 44.3% cell death at 80 *μ*M, which is the maximum for all the complexes. However, known anticancer drug Camptothecin showed 45.5% cell death at 6 *μ*M. Abundant evidences suggest that mitochondria plays a key role in the initiation of apoptosis by releasing Cytochrome C [44, 45]. In addition to Cytochrome C, other factors such as apoptosis signaling molecules and apoptosis inducing factor (AIF) can be important triggers of apoptosis [18]. It has been confirmed from cell cycle analysis (Figure 4) that cell death occurred through increase in hypo-diploid cells (Sub-G_1_ population) which indicates apoptosis. Previous studies showed that titanium affects polymerase proteins and transcription factors which inhibits protein synthesis and causes cytotoxicity [46].

## 4. Conclusions

We have reported the synthesis of mixed ligand titanium complexes having nitrogen containing ligands. The structure of the complexes has been confirmed by elemental analysis, FTIR, UV-visible, ^1^H NMR, and mass spectrometry techniques. Cytotoxic studies were done on different cell lines and it has been found that complex E with benzoylacetone ligand was a more potent cytotoxic agent. The morphological analysis on CHO cells indicates characteristics features of apoptosis and cell cycle analysis indicate increase in hypo-diploid cells. The mechanism of action has been certainly established *in vitro*; however, the efficacy of these complexes with their action mechanisms should also be demonstrated *in vivo*.

## Supplementary Material

Supplementary material: The FTIR spectra of ligands and titanium complexes are included.

## Figures and Tables

**Table 1:** Physical and analytical properties of titanium complexes.

Complex	Color	Yield (%)	M.P. (°C)	Experimental (Theortical) %	
				Ti	MW
Ti(ada)_2_(bpome)_2_ (A)	Cream	64.2	205–210	6.3 (6.1)	778 (780)
Ti(ada)_2_(dpme)_2_ (B)	Cream	87.3	205–210	7.1 (6.6)	713 (716)
Ti(ada)_2_(dach)_2_ (C)	White	87.2	225–230	8.3 (8.1)	574 (577)
Ti(ada)_2_(phen)_2_ (D)	Cream	87.5	210–215	6.0 (6.6)	705 (708)
Ti(ada)_2_(bzac)_2_ (E)	Light Orange	86	240–245	7.8 (7.1)	668 (670)

**Table 2:** Structure of ligands and proposed complexes.

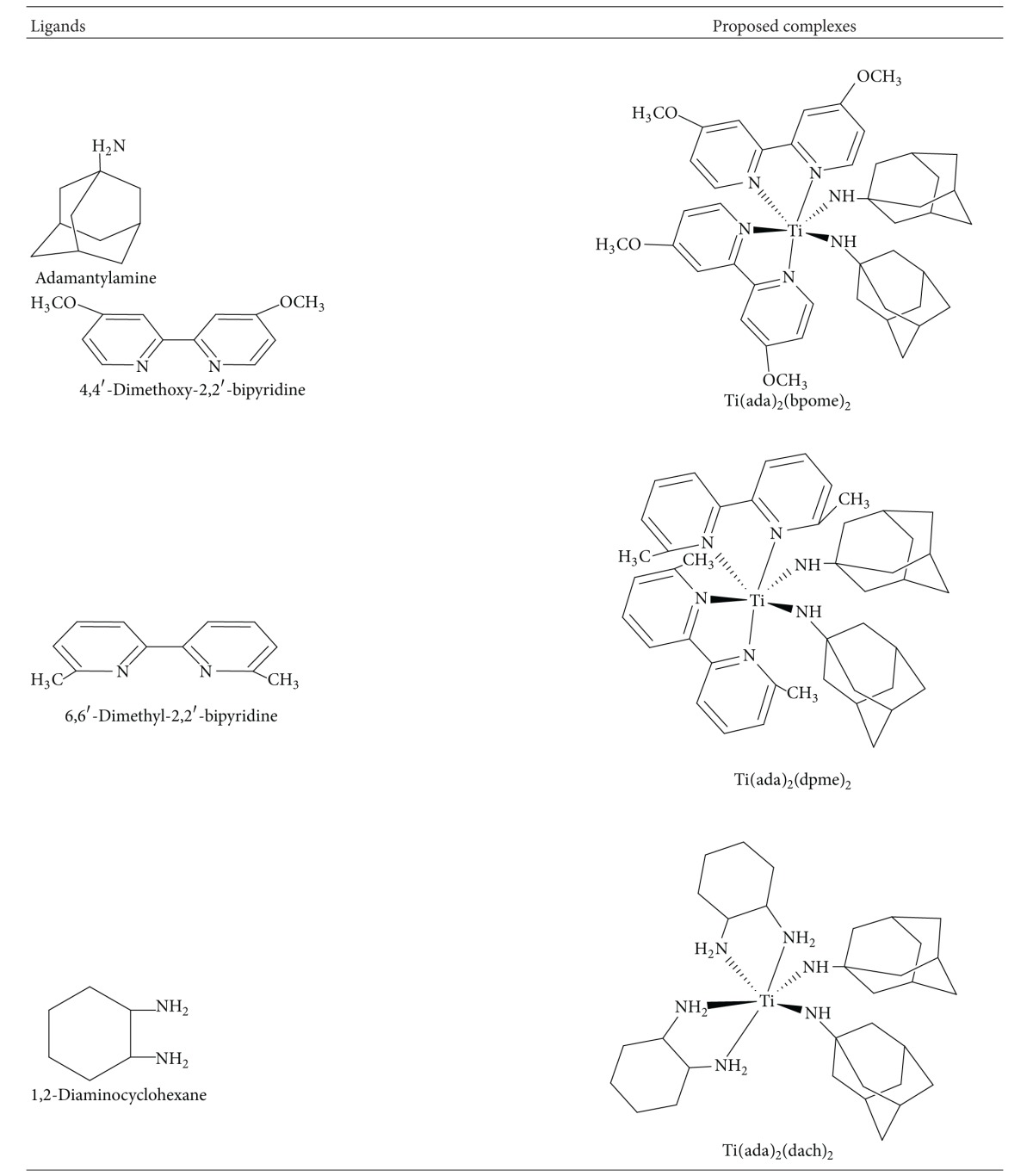 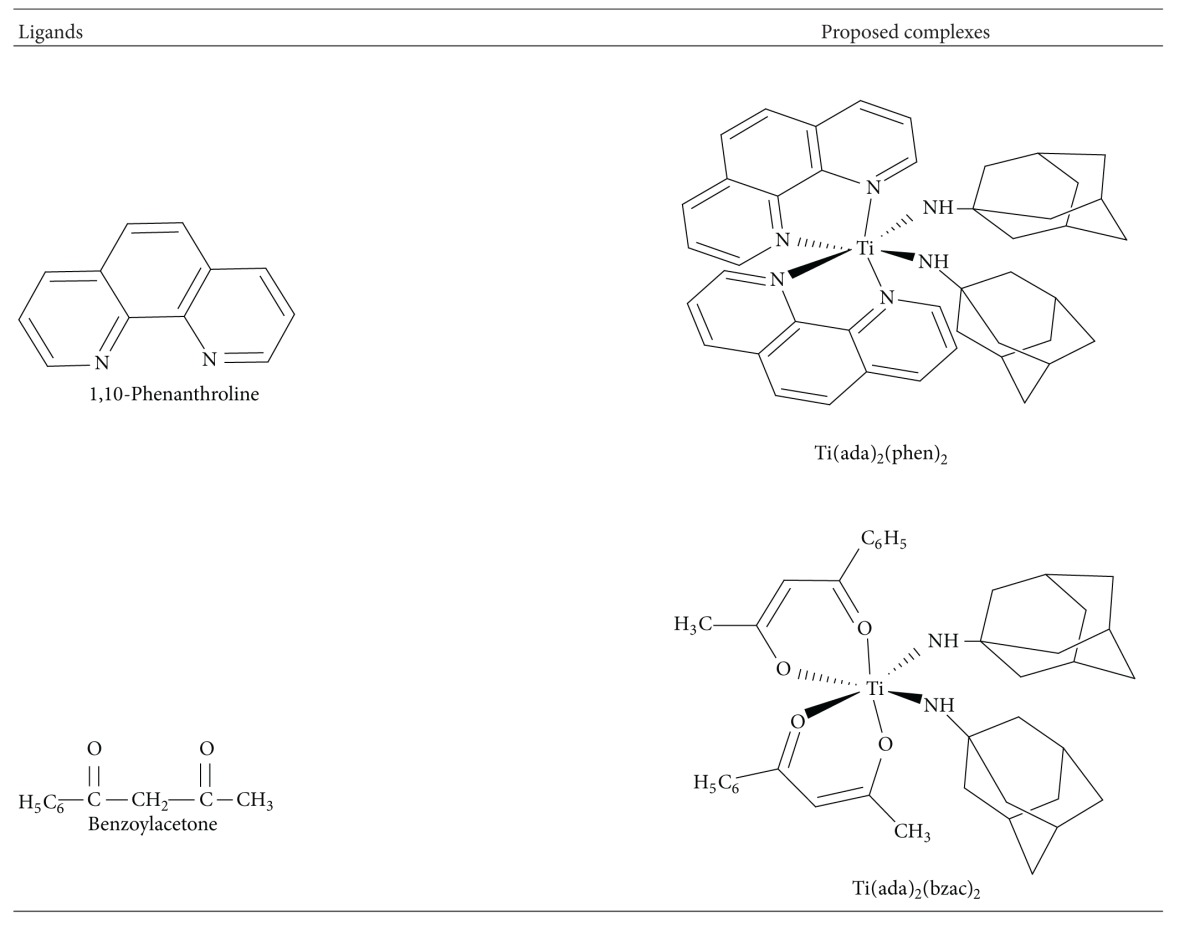

**Table 3:** Selected FTIR bands for titanium complexes and their corresponding ligands $\overset{-}{\nu }$ (cm^−1^).

Ligand/Complex	${\overset{-}{\nu }}_{\text{Ti–O}}$	${\overset{-}{\nu }}_{\text{Ti–N}}$	${\overset{-}{\nu }}_{\text{C–H}}$ bend	${\overset{-}{\nu }}_{\text{C–C/C=C}}$ stretch	${\overset{-}{\nu }}_{\text{N–H}}$ bend	${\overset{-}{\nu }}_{\text{C–H}}$ stretch	${\overset{-}{\nu }}_{\text{N–H}}$ stretch	${\overset{-}{\nu }}_{\text{C}=\text{O}}$ stretch
Ada	—	—	1132,1108	1457	1589	2912	3345,3372	
bpome	—	—	1303,1230	1595	—	3071,2974	—	
Ti(ada)_2_(bpome)_2_	—	452	1313,1229	1629	1449	3015,2927	3388	
dpme	—	—	1247,1158	1578	—	3063,2917	—	
Ti(ada)_2_(dpme)_2_	—	404	1271,1117	1644	1441	3071,2925	3336	
dach	—	—	1373,1072	1433	1578	2924	3357,3285	
Ti(ada)_2_(dach)_2_	—	444	1360, 1311	1470	1522	2925,2900	3379	
phen	—	—	1344,1093	1603	—	3055	—	
Ti(ada)_2_(phen)_2_	—	411	1368,1319	1612	1514	3039,2927	3412	
bzac			1255	1409		3063,3006		1603
Ti(ada)_2_(bzac)_2_	557	427	1109,1004	1522	1449	2933,2866	3379	1612

**Table 4:** XRD data of titanium complexes.

Empirical formula	TiC_44_H_54_N_6_O_4_ (A)	TiC_44_H_54_N_6_ (B)	TiC_32_H_58_N_6_ (C)	TiC_44_H_46_N_6_ (D)	TiC_40_H_48_N_2_O_2_ (E)
Formula weight	780	716	576	708	670
Crystal system	Monoclinic	Monoclinic	Monoclinic	Monoclinic	Monoclinic
Lattice type	P	P	P	P	P
*a* (A°)	17	13	16	11	17
*b* (A°)	13	11	12	13	11
*c* (A°)	14	20	15	14	16
*α* (°)	90	90	90	90	90
*β* (°)	91	106	113	106	85
*γ* (°)	90	90	90	90	90
Crystallite size (nm)	69	26.5	19.1	115	76.6
*V* (A°)^3^	3094	2860	2880	2002	2992
2*θ* start	10	10	10	10	10
2*θ* end	60	60	60	60	60
Radiation	Cu	Cu	Cu	Cu	Cu
Wavelength	1.54	1.54	1.54	1.54	1.54

**Table 5:** Cytotoxic studies of titanium complexes on HeLa, C6 and CHO cancer cell lines as determined by MTT assay.

Complex	Cell line (Source)		
	Hela (cervical)	C6 (Rat glioma)	CHO (Ovary)
	IC_50_ (µM)		
Ti(ada)_2_(bpome)_2_	13	17.8	19.9
Ti(ada)_2_(dpme)_2_	74	69.8	16.1
Ti(ada)_2_(dach)_2_	20.4	21	16.6
Ti(ada)_2_(phen)_2_	11.1	22.1	21.5
Ti(ada)_2_(bzac)_2_	4.06	21.8	46.1
Adamantylamine	104.5	148	123
Camptothecin	6.2	6.4	6.4
