# Supplementary material for: Synthesis, Spectral Characterization, and Antiproliferative Studies of Mixed Ligand Titanium Complexes of Adamantylamine
Source: Bioinorg Chem Appl. 2014 Feb 27;2014:142828. doi: 10.1155/2014/142828 (PMC3955668; doi:10.1155/2014/142828)
Supplement: Supplementary file 1 — Supplementary material: The FTIR spectra of ligands and titanium complexes are included. [file 142828.f1.docx]

**Supplementary data**

**FTIR spectra of ligands and titanium complexes**
